# Pleiotropic Phenotypes Associated With PKP2 Variants

**DOI:** 10.3389/fcvm.2018.00184

**Published:** 2018-12-18

**Authors:** Valeria Novelli, Kabir Malkani, Marina Cerrone

**Affiliations:** ^1^Centro Studi “Benito Stirpe” per la Prevenzione della Morte Improvvisa Nel Giovane Atleta, Institute of Genomic Medicine, Fondazione Policlinico Universitario Agostino Gemelli, Rome, Italy; ^2^Leon H. Charney Division of Cardiology, NYU School of Medicine, New York, NY, United States

**Keywords:** plakophilin-2, Arrhythmogenic Cardiomyopathy, ARVC, Brugada syndrome, sudden cardiac death, genetic mutation, cardiomyopathies

## Abstract

Plakophilin-2 (PKP2) is a component of the desmosome complex and known for its role in cell-cell adhesion. Recently, alterations in the *Pkp2* gene have been associated with different inherited cardiac conditions including Arrythmogenic Cardiomyopathy (ACM or ARVC), Brugada syndrome (BrS), and idiopathic ventricular fibrillation to name the most relevant. However, the assessment of pathogenicity regarding the genetic variations associated with *Pkp2* is still a challenging task: the gene has a positive Residual Variation Intolerance Score and the potential deleterious role of several of its variants has been disputed. Limitations in facilitating interpretation and annotations of these variants are seen in the lack of segregation and clinical data in the control population of reference. In this review, we will provide a summary of all the currently available genetic information related to the *Pkp2* gene, including different phenotypes, ClinVar annotations and data from large control database. Our goal is to provide a literature review that could help clinicians and geneticists in interpreting the role of *Pkp2* variants in the context of heritable sudden death syndromes. Limitations of current algorithms and data repositories will be discussed.

## Introduction

The recent expansion of genetic testing in the field of cardiac arrhythmias, due to decreased sequencing costs and increased availability of large comprehensive panels, recently brought up the discoveries that variants in the same genes can be detected in association with different phenotypes. The pleiotropic effect of a gene, i.e., its potential to influence different clinical phenotypes, reflects the new notion that the same protein could exert different and unrelated functions, in addition to the one historically associated with it. On the other hand, the increased number of variants discovered in association with multiple phenotypes and often without robust linkage data raised, in parallel, concerns on the evidence supporting their genotype-phenotype causal relation.

As an example of pleiotropic genes, we are focusing our review on plakophilin-2 (PKP2) coded by the gene *Pkp2*, whose pathogenic role has been recently recognized in different inherited cardiac arrhythmias syndromes, ranging from Arrhythmogenic Cardiomyopathy (ACM or ARVC), Brugada Syndrome (BrS), idiopathic ventricular fibrillation, hypertrophic cardiomyopathy (HCM), and dilated cardiomyopathy (DCM). Evidence has simultaneously emerged of the positive Residual Variation Intolerant Score (RVIS) (1) of *Pkp2* suggesting that its overall variation could be quite tolerated. Along these lines, a study showed that several *Pkp2* variants can be found in a high percentage of ostensibly healthy controls, adding to the complexity of interpretation of the genetic variability of this protein (2). We will review the spectrum of phenotypes that have been attributed to *Pkp2* variants and discuss clinical and functional evidence, as well as controversial interpretations in light of current data deriving from large statistical analyses and public databases.

## Interpretation of Genetic Variants: Large Population Database

Currently, the adjudication of the clinical significance of genetic variants is among the major limitations of genetic testing (3). The increasing progress in sequencing technology unraveled the complexity of human genetic variability, bringing up the new challenge of distinguishing between variants with a potential deleterious effect and the ones which may only be a bystander.

Several public databases have been created with the goal of facilitating this task. The most cited annotation database is ClinVar (https://www.ncbi.nlm.nih.gov/clinvar/) (3). This database is part of the NCBI's Entrez system, and includes information for more than 30,181 genes. This is the first freely available archive of reports from several selected sources which attempts to establish a relation between variants and phenotype. ClinVar accepts direct submissions from genetic laboratories, academic centres, genetic repositories, and scientific societies that are required to share the phenotype details, the methodology used to capture variant calls and finally their clinical interpretation. However, there is a lack of structured determination, since the respective submitter determines the variant adjudication in ClinVar, based on internal criteria and possibly in agreement with the American College of Medical Genetics (ACMG) guidelines. Additional limitations are the lack of a central review process, the scarce if any clinical data provided and the lack of a defined denominator, since the latter is established by each single submitter based on their own screening panel features and on the number of panels and clinical diagnoses encompassing a specific gene. Hence, the integration with additional independent sources is pivotal for an appropriate interpretation of data in ClinVar in the clinical context.

In the past few years, several databases of whole exome data from large populations of ostensibly healthy controls have been made available to the public. The goal of this repositories is to provide information on the expected human variation frequency. But the lack of phenotypical data may create controversial interpretation even when using these sources. The Genome Aggregation database (GnomAD; gnomad.broadinstitute.org) is currently one of the largest available and the most used support for variant interpretation. This freely accessible database includes genetic data from 123,136 exome sequences and 15,496 whole-genome sequences. It is mainly used to discriminate common from rare variants, across eight different ethnic groups. Usually, a Minor Allele Frequency (MAF) >0.001 is considered the threshold to discriminate between common from rare variants, although the value should be adjusted by disease prevalence, which is often a challenge to determine, especially in the case of cardiac channelopathies and cardiomyopathies that show incomplete penetrance and concealed or progressive phenotypes.

A new statistically robust framework to assess disease-specific thresholds has also been recently developed. This support, freely available at http://cardiodb.org/allelefrequencyapp/ (4) allows to calculate the maximum expected allele frequency of a disease-causing variant in the general population in order to classify it according to the phenotype. The algorithm has been created in order to address the arbitrarity of the cutoff determination in mendelian disorders. It combines disease prevalence, genetic and allelic heterogeneity, inheritance mode, penetrance, and sampling variance in reference database to assess the frequency cutoff. In the initial presentation of their algorithm, the authors show a false positive rate < 0.001 when applied to HCM-related variants. The application of this new algorithm to *Pkp2* in the future may shed some light of the expected pathogenicity of several unresolved variants at least for the ACM phenotype.

These tools combined together represent a helpful “first-pass” interpretation that should be followed by a detailed analysis from genetic experts taking into account the clinical presentation of the patient and evidence of co-segregation in the family.

## Plakophilin-2: Structure and Functions

Desmosomes are intercellular junctions of epithelia and cardiac muscle (5), tethering intermediate filaments to the plasma membrane to maintain cell adhesion and tissue integrity. Structurally, they are composed by three major gene families: desmosomal cadherins, armadillos, and plakins (6).

The *Pkp2* gene has been characterized for the first time in 1996 (7) and encodes for the PKP2 protein. The main recognized function of PKP2 is mechanical, i.e., providing a lateral stabilizing force with the desmosomal-intermediate filament assembly facilitating cell-to-cell contact. However, in recent years evidence has been provided of the pleiotropic role of this protein with functions ranging from intracellular signaling regulation to electrophysiologic and trafficking regulation, to the control of transcription processes. Studies oriented to discover the relation between defective PKP2 and arrhythmias showed that PKP2 is necessary to maintain gap junction integrity and formation (8–10): indeed, hearts samples from ACM patients presented loss of gap junction plaques (11). Subsequently, it was demonstrated that loss of PKP2 expression disrupts trafficking of the sodium channel at the intercalated disc, thus decreasing cardiac sodium current and facilitating arrhythmias (12, 13). These effects reflect the interplay between defective PKP2 and the molecular complex at the intercalated disc defined “connexome”(14, 15).

More recently, data on a novel cardiac-specific, tamoxifen activated PKP2 knock-out mouse model linked loss of PKP2 to transcriptional disruption of calcium homeostasis (16). In addition to these electrophysiologic effects, it has been proposed that PKP2 deficiency could activate the Hippo and Wnt pathways, facilitating fibro-adiposis in ACM (17, 18). Dubash et al. (19) showed another mechanism by which PKP2 may facilitate fibrosis formation: in their work, lack of PKP2 expression caused increased expression of TGF-beta1 and activation of the p38-MAPK-dependent pro-fibrotic program.

The different functions and protein-protein interactions that have been demonstrated for PKP2 provide interesting insights on the complexity of proteins functions that often overcome the primary role attributed at the time of initial discovery and could justify some of the pleiotropic phenotypical manifestations of its genetic variants.

## PKP2 Variants in ACM

ACM is a familial disease characterized by ventricular arrhythmias, increased risk of sudden cardiac death (SCD) and progressive fibrofatty replacement of the myocardium, usually starting at the right ventricle and subsequently evolving to biventricular dilation and heart failure (20). The symptoms usually manifest starting from early adulthood and the disease has an estimated prevalence of 1:2,000–1:5,000 in the general population, is more common in males (2:1, which also seems to show higher incidence of fatal arrhythmic events (20, 21). However, the disease phenotype is highly variable and characterized by incomplete penetrance. SCD can be its initial manifestation even before an overt cardiomyopathy is evident (20).

Currently, 14 different genetic loci have been reported for ACM, the majority being desmosomal genes such as *Pkp2, Dsp*, and *Dsg2 and Dsc2*. In a minority of the cases, alterations in other structural cardiac genes have been also detected (22). Furthermore, compound/digenic heterozygosity has been identified in about 4% of ACM mutation carriers (21). Data on patients with compound/digenic heterozygosity suggest that they might show an earlier onset of symptoms (21). Genetic alterations in *Pkp2* are the most frequent, accounting for 40–60% of the genotype positive patients (20, 23, 24). These include single amino acids mutations such as missense, non-sense or frameshift variants, but also large genomic rearrangements of several exons (23, 25).

At present, the ClinVar database (accessed June 2018) would return a total of 521 annotated *Pkp2* variants. While the majority (65%) is linked to a cardiac condition, the remaining 35% has been submitted without an associated clinical diagnosis. A search only for “ACM” yielded 290 alterations. Among those, 83 (29%) are classified as pathogenic (P) /likely pathogenic (LP), 66 (24%) are benign (B)/likely benign (LB) and 110 are uncertain significant (VUS) (38%). In addition, about 10% (26) of the total reported variations in ClinVar has a conflicting interpretation based on discordant data among different reports (Table 1).

**Table 1:** *PKP2* variants reported in ClinVar related to different cardiac conditions.

**Condition**	**P/LP**	**VUS**	**B/LB**	**Conflicting interpretation**
ACM	83	110	66	31
CV phenotype	6	19	4	2
Cardiomyopathy	2	1		
HCM		1		1
DCM	1			
VF				1
VT		2		
LVNC		1		

*P, Pathogenic; LP, Likely Pathogenic; VUS, Variant with uncertain significance; B, Benign; LB, Likely Benign; ACM, Arrhythmogenic Cardiomyopathy; CV, Cardiovascular; HCM, Hypertrophic Cardiomyopathy; DCM, Dilated Cardiomyopathy; VF, Ventricular Fibrillation; VT, Ventricular Tachycardia; LVNC, Left Ventricular Non Compaction*.

If one focuses on P/LP variants, only 5 of them (6%) are missense, whereas most are non-sense (25%), frameshift variants (46%), splicing alterations ±1 or ±2 (17%) or large deletions (6%). In contrast with this finding, among the ones classified as variant of unknown significance (VUS) 59% are missense, 12% are intron variants located relatively far from the donor or acceptor sites, 3% are synonymous variants and 25% are nucleotide substitutions in the 3′UTR. This data shows that the majority of *Pkp2* variants (~77%) associated with ACM in ClinVar and annotated as P/LP are radical alterations, hence considered at high probability of causing a disruption of the protein and resulting in extensive transcriptional and posttranslational alterations, while single amino acid changes are of more complex interpretation, especially in light of the known “signal-to-noise ratio” (2).

However, according to the ACMG and the Association of Molecular Pathology (27) guidelines, the type of variant is only one among the criteria considered useful to adjudicate pathogenicity. Another relevant criterion, is the analysis of the variant frequency in the general population (4). When analyzing the allele frequency of the ACM-related 83 P/LP mutations from ClinVar, only 19 (23%) of these are also present in GnomAD (June 2018), hence in apparently healthy controls. These are all reported with an allele frequency ranging 0.000004–0.00004 as shown in Table 2. Considering that ACM is a rare autosomal disease, with adult onset and incomplete penetrance, a very low allele frequency is not unexpected, thus their presence in GnomAD does not necessarily rule out a pathogenic role. The remainig 77% of P/LP ACM variants from ClinVar are not reported in GnomAD, in support of their potential causative disease role. In contrast with the data for P/LP mutations, 61% of *PKP2* VUS annotated in ClinVar are also present in GnomAD and their allele frequency ranges 0.00003–0.001. This higher allele frequency, which encompasses also synonymous and UTRs variants, far from the flanking regions, adds on the controversial interpretation of their effect.

**Table 2:** Allele frequency of the ACM-related Pathogenic/Likely Pathogenic variants reported in ClinVar according to GnomAD (both database accessed on June 2018).

**Variants**	**#rs**	**Allele count**	**Allele frequency**
c.2489+1G>A	rs111517471	6	2,17E-05
p.Arg735Ter	rs121434421	1	4,06E-06
c.2146-1G>C	rs193922674	10	3,61E-05
p.Glu667Ter	rs397517015	1	4,06E-06
p.Arg651Ter	rs751288871	2	7,22E-06
p.Gln638Ter	rs397517012	2	7,22E-06
c.1688+1G>A	rs397517003	1	4,07E-06
p.Trp538Ter	rs193922672	3	1,22E-05
c.1378+1G>C	rs397516994	1	4,07E-06
p.Arg413Ter	rs372827156	4	1,44E-05
p.Arg388Trp	rs766209297	1	4,06E-06
p.Gln378Ter	rs397516986	2	7,22E-06
p.Tyr221Ter	rs767987619	2	7,22E-06
p.Trp123Ter	rs774663443	1	4,12E-06
p.Leu771ProfsTer2	rs121434420	1	4,06E-06
p.Arg79Ter	NA	1	4,07E-06
p.His318TrpfsTer10	NA	1	4,07E-06
p.Gln323ArgfsTer12	rs745882420	13	4,71E-05
p.Val280HisfsTer55	rs772220644	2	7,23E-06

In summary, large population databases support the established relation between *Pkp2* and the ACM phenotype and confirm published evidence that radical mutations are often associated with a more severe clinical presentation (2, 27).

## PKP2 and Brugada Syndrome

Genetic variants in *Pkp2* have also been recently identified in patients affected by BrS (28). This inherited arrhythmia is characterized by ST- segment elevation in the right precordial leads, and increased risk of ventricular fibrillation, without macroscopic structural disease (29). Over 22 different genes have been so far linked to BrS (30). Mutations on the SCN5A gene, coding for the alpha-subunit of the cardiac sodium channel account for ~25% of cases and all other genes cover altogether only 5–10% of genotyped patients (31). Polygenic inheritance has also been suggested (26). Interestingly, past studies have suggested a possible overlap between the BrS and ACM phenotypes, suggesting that the two diseases could be at the opposite end of a common clinical condition (32). Functionally, *SCN5A* loss of function variants associated with BrS lead to decreased sodium current, through different mechanisms (29, 31).

Following a gene-candidate approach based on the relation between lack of PKP2 expression and decreased sodium current *in vitro*, we reported the first cases of patients with a BrS phenotype in the absence of overt structural cardiomyopathy carrying genetic variants in the *Pkp2* gene (28). We discovered five different *Pkp2* missense variants in five unrelated individuals, and provided genotype/phenotype co-segregation data in one family across two generations. Functional *in vitro* studies in HL1 cardiac cells and in human ips-derived cardiomyocytes demonstrated that these variants could decrease sodium current, an electrophysiologic effect consistent with the BrS phenotype, thus supporting the potential pro-arrhythmic role of the variants. Based on this initial study, followed by other clinical case reports (33), some commercial genetic panels for BrS now include *Pkp2*.

Due to its recent inclusion in commercial testing, ClinVar does not yet report any *Pkp2* variant under the “BrS” diagnosis. At present there have been only 8 PKP2 non-synonymous variants associated with BrS reported in the literature (28, 33, 34). Three of these are absent in ClinVar, and the remaining 5 are VUS or have a “conflicting interpretation” for the diagnoses “ACM” or “cardiovascular phenotype” (Table 1). GnomAD offers contrasting interpretation in respect from ClinVar for these 8 variants, whose allele frequency ranges from 0.0002382 to 0.000008127, hence supporting a possible pathogenic role.

However, in this perspective it is important to highlight data from a recent large study that challenged the role for “minor” BrS genes, including *Pkp2*, as causal for BrS (35). The authors used an evidence-based semiquantitative scoring system of genetic and experimental evidence, which showed that only the gene SCN5a could be linked to the disease with a definite evidence.

## Additional Cardiac Phenotypes Linked to PKP2

The availability of large panels and of exome screening has allowed investigators to discover pathogenic variants in genes initially unexpected to be related to the phenotype. In the case of *Pkp2*, variants have been detected in idiopathic ventricular fibrillation and SCD (36, 37), possible catecholaminergic polymorphic ventricular tachycardia (38), HCM, DCM and left ventricular non-compaction (39–41). The association between *Pkp2* mutations and idiopathic SCD is not that surprising, considering that often SCD is the earliest manifestation in ACM athletes before the onset of overt cardiomyopathy. The association between Pkp2 variants and other structural cardiomyopathies aside from ACM remains instead quite controversial, as discussed further. A ClinVar search for PKP2 variants based on following diagnoses: “cardiovascular phenotype,” “cardiomyopathy,” “LVNC,” “Paroxysmal familial ventricular fibrillation,” “DCM,” “HCM,” and “ventricular tachycardia,” returns 41 different changes. However, most of them (76%) appear under the generic definition of “cardiovascular phenotype,” which is not an informative diagnosis. By narrowing the search to DCM and HCM, ClinVar reports 3 variants only (1 DCM and 2 HCM) annotated as “LP” (H679T), “VUS” (M349V) and “conflicting interpretation” (T526A), respectively (Table 1). Among these, the two that are associated with HCM are reported in GnomAD with an allele frequency of 0.00012 and 0.000036, respectively. The relation between non-sarcomeric genes and HCM has been questioned recently in a large statistic-based approach study (42), showing limited evidence that these genes associate with the phenotype. Even if *Pkp2* was not included in the study, the data suggest caution in the evaluation of these reported variants.

No data regarding the variant associated with DCM is reported in GnomAD, supporting its low prevalence. However, if this is a variant linked to a clinical phenotype that initially manifested as ACM and then evolved into DCM is yet to be assessed. Considering that clinically cardiomyopathies are diseases with a progressive course, one cannot exclude that DCM cases carrying *Pkp2* variants could be cases of advanced ACM which were missed in the initial disease phases.

The 2 PKP2 VUS linked to “Ventricular Tachycardia” (Q59L and G327V) have a low allele frequency in GnomAD (0.00002 and 0.00009, respectively). The splice site alteration (c.1974A>G) for the ClinVar diagnosis of “paroxysmal ventricular fibrillation” (conflicting interpretation) is not present in GnomAD.

## Conclusions

The expansion and increased availability of genetic testing has challenged the concept of “one gene-one disease” and has shown that different phenotypes can be caused by variants on one same gene. The interpretation of these findings in light of human variation data is complex and casts some warning on the clinical application of this information. The evidence of the pleiotropy of a gene suggested by genetic variants and their functional effect *in vitro* has the important value of discovering different protein functions and suggest arrhythmia mechanisms. The use of all this data for clinical diagnosis and risk assessment may not yet be ready for prime time, as shown by recent large statistical studies. Current database and *in silico* tools have known limitations, but the combination of this evidence with increasing collaborative efforts pooling clinical and genetic information together could in the future shed new light on how to interpret these data in lights of patients' care.

## Author Contributions

VN: Initial preparation of the manuscript and figures; KM: Critical revision of article; MC: Article conception and design, critical revision of manuscript, approval of final version.

### Conflict of Interest Statement

The authors declare that the research was conducted in the absence of any commercial or financial relationships that could be construed as a potential conflict of interest.
